# Global Genetic Variation in Circulating 25-Hydroxyvitamin D: A Systematic Review of GWAS Evidence Across Different Ancestral Groups

**DOI:** 10.3390/nu18132052

**Published:** 2026-06-24

**Authors:** Alexandros Papoutsis, Danae Malikides, Andrea Georgiou, Demetris Lamnisos, Alexandros Heraclides

**Affiliations:** 1 Department of Health Sciences, School of Life and Health Sciences, European University Cyprus, 2404 Nicosia, Cyprus; 2 Department of Psychology, University of Cyprus, 1678 Nicosia, Cyprus

**Keywords:** vitamin D deficiency, 25-hydroxyvitamin D, genome-wide association study, genetic variation, ancestry, linkage disequilibrium, systematic review

## Abstract

Background/Objectives: Vitamin D deficiency is a global health concern, yet circulating 25-hydroxyvitamin D (25OHD) concentrations vary substantially across geographical regions and ancestral groups. Genetic predisposition may contribute to these differences. This systematic review aimed to synthesize evidence from genome-wide association studies (GWAS) on genetic variation associated with circulating 25OHD across populations from different ancestral backgrounds and to evaluate linkage disequilibrium (LD) between reported variants. Methods: A systematic review was conducted according to PRISMA 2020 guidelines. PubMed and the GWAS Catalog were searched to identify genome-wide association studies (GWAS) on circulating 25-hydroxyvitamin D (25OHD) concentrations. Studies were screened against predefined eligibility criteria, and data were extracted using a standardized framework. Methodological quality was assessed using a standardized tool, and study power adequacy was assessed formally. Genome-wide significant SNPs were extracted, and unique variants between studies were grouped by ancestry. Among these, dbSNP-indexed variants were grouped into genomic cluster windows and evaluated for LD structure. Results: Fifteen GWAS were included. Across these studies, 349 genome-wide significant SNP associations were identified, corresponding to 294 unique variants, of which 283 were indexed in dbSNP and retained for genomic and LD analyses. Variant discovery was dominated by large-scale European-ancestry studies, although African, Middle Eastern, East Asian, Hispanic/Latino, South Asian, and trans-ethnic studies also contributed signals. Some evidence of ancestry-specific variation was apparent, yet not conclusive due to lower study power in non-European cohorts. Variant aggregation was strongest at biologically relevant vitamin D loci, including *GC*, *CYP2R1*, *DHCR7*/*NADSYN1*, and *FLG*. Fifteen variants were replicated in at least two independent cohorts. LD-based clustering identified several high LD groups comprising variants identified across studies, with the strongest LD appearing between variants within established vitamin D-related loci, particularly *GC*, *CYP2R1*, *DHCR7*/*NADSYN1*, and *FLG*. Conclusions: Circulating 25OHD appears to be influenced by shared core loci involved in vitamin D metabolism, across ancestries. Although some evidence of ancestry-specific variation was identified, findings should be interpreted with caution, in light of the predominance of European-ancestry GWAS and scarcity of sufficiently powered GWAS for other ancestral populations. Larger GWAS in non-European populations are essential for improving ancestry-specific variant discovery and interpretation.

## 1. Introduction

Vitamin D deficiency (VDD), typically defined as serum 25-hydroxyvitamin D (25OHD) levels < 50 nmol/L (<20 ng/mL), and insufficiency ranging between 50 and 75 nmol/L (20–30 ng/mL) [1] is a global health concern. Factors contributing to deficiency include limited sun exposure due to geographical location, lifestyle and cultural factors such as indoor living and modern work environments, excessive sunscreen and clothing use [2], as well as poor diet and obesity [3]. Addressing vitamin D deficiency is particularly important in regions with high prevalence rates, as it can help reduce the burden of related health conditions and improve overall population health [4].

Consistent evidence supports an important role of vitamin D in skeletal health among vitamin D-deficient and older individuals, although benefits in the general population are less clear [5]. Beyond skeletal outcomes, evidence deriving primarily from observational studies implicating low vitamin D levels to a multitude of chronic conditions, including cardiometabolic disease and cancer [6,7,8], has been recently refuted by randomized controlled trials and Mendelian Randomization analyses [9]. Mendelian randomization studies provide evidence supporting causal associations between low vitamin D status and only selected outcomes such as all-cause mortality and multiple sclerosis [10,11,12,13]. RCTs and umbrella reviews are also in agreement regarding a general lack of association between low vitamin D levels and several non-skeletal outcomes, yet interventional studies do show the potential benefits of supplementation as regards cancer mortality [14,15,16], overall mortality among critically ill patients [15,17,18], and improvements in the quality of life among diseased populations [19].

Substantial variation in circulating 25OHD concentrations is observed across populations worldwide [20]. The global variation in vitamin D levels across geographical regions and ancestral groups is substantial, highlighting a paradox in global vitamin D status. Northern European countries, despite limited sun exposure, generally demonstrate adequate population-level vitamin D concentrations [21], while in regions with abundant sunlight, including North Africa, the Eastern Mediterranean, and the Middle East, high rates of vitamin D deficiency are reported in the literature [20]. Systematic reviews in the Middle East and North Africa (MENA) region consistently report high prevalence VDD (~43%), with women exhibiting significantly lower mean vitamin D concentrations than men [22]. Remarkably high rates of deficiency/insufficiency have been reported in specific regions with an abundance of sunshine, such as Greece (72.7%) and Cyprus (69.3%) [23], Lebanon (~70.0%) [24] and Israel (~50.0% in the general populations and up to 76.7% among Arab sub-populations) [25,26,27].

Similarly, vitamin D deficiency is particularly widespread in South Asian populations. A pooled prevalence of approximately 68% has been reported across South Asia, with a marked sex disparity, with women being disproportionately affected (76%) compared to men (51%) [28,29,30]. The same picture has been observed in populations of African ancestry, despite high ambient ultraviolet radiation, largely attributed to increased skin melanin content, limited dietary vitamin D intake, and socioeconomic factors [29].

This paradox, characterized by lower vitamin D levels in sunnier regions, warrants further investigation. While sun exposure, dietary intake, and cultural practices are significant determinants of vitamin D status, genetic predisposition might be equally important. Possible explanations stemming from different evolutionary pressures resulting in beneficial genetic variation, as regards higher 25OHD concentrations have been proposed. Following the out-of-Africa expansion into Eurasia, between 50,000 and 75,000 years ago, there was an essential need for additional adaptation to northern latitudes and the lower exposure to UVB radiation along the route out of Africa [30], potentially involving more efficient synthesis of vitamin D [31]. There is clear evidence of pigmentation genes [31,32] and other genetic variants involved directly or indirectly in vitamin D synthesis and metabolism, with a positive selection in Europeans and surrounding populations [33,34]. A clear example of the important role of ancestry-specific genetic architecture in determining vitamin D levels is the consistently observed lower 25OHD concentrations observed among Southeast Asian and African decent individuals residing in Europe, compared to the white European population [35,36,37].

Although numerous GWAS have identified genetic variants associated with circulating 25OHD concentrations, the available evidence remains dispersed across studies conducted in different populations and ancestry groups. To date, there has been no comprehensive synthesis of GWAS findings examining the overall genetic architecture of circulating 25OHD concentrations across diverse ancestral backgrounds while also evaluating the degree of overlap and independence between reported loci.

An additional challenge in interpreting GWAS findings is that different studies may report different lead variants within the same genomic region. Such variants may represent the same underlying association signal because of linkage disequilibrium, or they may indicate distinct independent signals within a broader locus [38]. Evaluating LD among reported variants can therefore help distinguish recurrent proxy signals from potentially independent loci, which is particularly relevant in cross-ancestry comparisons, where allele frequencies and LD structure may differ between populations.

This systematic review aimed to detect and synthesize the available evidence from genome-wide association studies on genetic variation associated with circulating 25OHD across populations from different ancestral backgrounds. A second aim was to address the cross-study linkage disequilibrium between variants reported in independent GWAS and identify independent ancestry-specific loci across studies.

## 2. Materials and Methods

This systematic review was conducted and reported in accordance with the PRISMA 2020 Expanded Checklist, adapted for genome-wide association studies. Several items related to intervention effects, quantitative meta-analysis, and conventional risk-of-bias tools were not applicable, due to the observational and genomic nature of the included studies. Instead, the methodological quality and comparability were assessed through key GWAS-specific criteria, including population stratification control, phenotype transformation, replication strategy, and linkage disequilibrium handling.

The review protocol was not prospectively registered. The review was conducted using predefined eligibility criteria, standardized data-extraction procedures, and prespecified approaches for variant grouping, genomic clustering, LD assessment, methodological quality assessment, and power appraisal, and was reported in accordance with PRISMA 2020.

### 2.1. Search Strategy

The systematic review was conducted using two databases, PubMed and the GWAS Catalog, to identify eligible genome-wide association studies investigating circulating 25OHD concentrations. No publication date restrictions were applied. The search strategy combined established GWAS-related terms (“genome-wide association”, “genomewide association”, and “GWAS”) with commonly used vitamin D-related terms (“25-hydroxyvitamin D”, “25 hydroxyvitamin D”, “25(OH)D”, “25-OH-D”, “25OHD”, and “calcifediol”). The literature search was updated on 12 June 2026 prior to manuscript resubmission to identify any additional eligible studies. The complete PubMed search strategy is presented in Supplementary Table S1.

The GWAS Catalog does not allow for systematic literature searches using conventional Boolean operators. Therefore, studies were identified using the trait term “vitamin D amount” together with the relevant database filtering options available at the time of access. In particular, studies ‘with full summary statistics’ as reported in the GWAS Catalog, were selected. The GWAS Catalog search (updated on 12 June 2026) procedure, including trait terms, filters, and date of access, is provided in the Supplementary Materials. This search identified 73 genetic association analyses, of which 15 fulfilled the criteria for a GWAS.

### 2.2. Inclusion and Exclusion Criteria

The primary inclusion criterion was studies classified as GWAS, reporting genetic variation linked to circulating 25OHD concentrations. The exclusion criteria included systematic reviews, non-GWAS studies (e.g., candidate-gene association studies), studies in which 25OHD was not among the study outcomes, and studies not published in English. Studies employing a GWAS design were additionally excluded when their sample size was substantially below the minimum requirements for adequate statistical power at the conventional genome-wide significance threshold (*p* < 5 × 10^−8^). Power adequacy was evaluated under multiple scenarios reflecting different combinations of allele frequency and effect size, and studies with sample sizes insufficient to detect even high-frequency variants with strong effects were classified as small/pilot GWAS and excluded.

### 2.3. Study Selection

**Figure 1:**
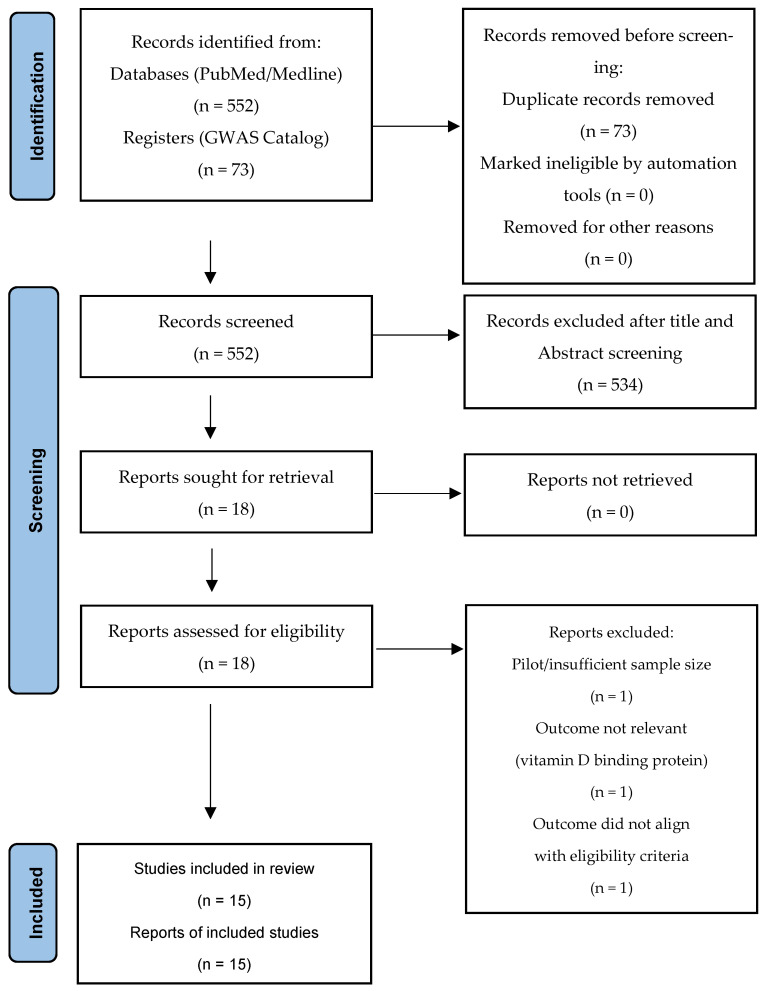
Flow diagram for study inclusion in the review following the PRISMA guidelines.

A comprehensive screening procedure was undertaken in accordance with the study’s predefined inclusion and exclusion criteria. Title and abstract screening, full-text eligibility assessment, data extraction, and methodological quality assessment were conducted independently by two reviewers using predefined eligibility criteria and a standardized data extraction framework. Any discrepancies between the two review processes were evaluated and resolved through discussion within a three-member review team consisting of the two reviewers and the senior author/group leader. The independent assessments produced highly consistent results, with only one additional eligible study identified in one review process (Hendi et al., 2025) [39]. The initial search yielded 625 records (552 sourced from PubMed and 73 from the GWAS Catalog). Evaluation of duplicates revealed that all 73 studies identified via the GWAS Catalog were also provided via the PubMed search. After duplicate removal, 552 unique articles proceeded to the first stage of evaluation, during which titles and abstracts were examined to determine their potential relevance. Only studies that appeared to meet the eligibility requirements advanced to full-text review. This second stage resulted in 18 articles being assessed in detail. Following closer examination, 3 of these were excluded: one due to an insufficient sample size and two because the reported outcomes did not align with the criteria. Consequently, 15 studies were deemed suitable for inclusion in the final systematic review. To visually document each step of this selection pathway, the process was mapped using the Preferred Reporting Items for Systematic Reviews and Meta-Analyses (PRISMA) flow diagram, presented in Figure 1. The completed PRISMA 2020 checklist is provided in the Supplementary Materials (Supplementary Table S13).

### 2.4. Data Extraction

For each eligible GWAS, data were extracted using a standardized data extraction framework. The extracted study-level variables included the first author, publication year, sample size, ancestry group, phenotype definition and transformation, genotyping platform, GWAS analytical approach, population structure adjustment strategy, and covariates included in the analysis. Variant-level information included the SNP identifier (rsID), genomic position, effect allele, non-effect allele, effect size estimate, standard error (where available), *p*-value, effect allele frequency (where available), and direction of effect. Additional information regarding conditional analyses, confounding adjustment, signal selection procedures, and replication strategies was also extracted where reported. Only effects meeting the conventional genome-wide significance threshold (*p* ≤ 5 × 10^−8^) were retained. The complete catalogue of extracted study-level and variant-level information is provided in Supplementary Table S3, which corresponds to the standardized data extraction worksheet used during the review process.

### 2.5. Quality Assessment

Methodological appraisal of the included studies was conducted using a series of GWAS-relevant methodological considerations across studies (Supplementary Table S2).

Furthermore, the power sufficiency of the included GWAS was evaluated by comparing each study’s reported sample size against sample size requirements derived in R using the genpwr package (v1.0.4) under a linear regression framework with an additive genetic model 80% power and the conventional genome-wide significance threshold of α = 5 × 10^−8^. Two allele-frequency scenarios were selected: MAF = 0.05, representing variants at the lower boundary of common-variant GWAS discovery where power is more limited, and MAF = 0.30, representing common variants with higher expected power. Three standardized per-allele effect-size scenarios were evaluated: β = 0.03, 0.10, and 0.30 SD units, intended to represent weak, moderate, and strong genetic effects, respectively. These values were not intended to reflect study-specific observed effects but to provide a consistent benchmark for comparing the approximate power profile of studies with very different sample sizes.

For each allele-frequency/effect-size combination, the required sample size was compared with the reported analysis sample size of each GWAS. Studies were classified as sufficiently powered for a given scenario when their sample size met or exceeded the required sample size threshold; studies that did not meet the threshold were classified as underpowered for that scenario. Where a study’s sample size was close to but below the required threshold, this was labelled as borderline. The resulting classification was used to summarize the breadth of power adequacy across predefined scenarios and is presented in Supplementary Table S2.

### 2.6. Data Synthesis of Reported Effects

Given the substantial heterogeneity in phenotype assessment phenotype transformation, reporting format, and analytical approaches across included GWAS, a formal meta-analysis was not undertaken. Instead, the results were synthesized using a structured qualitative and comparative framework. Differences in 25OHD measurement (e.g., assay type, and unit of measurement) and transformation for analysis (e.g., raw, log-transformed, standardized, square-root, or rank-based inverse normal transformation) were explicitly documented and considered during interpretation by classifying studies according to outcome scale. Reported effect estimates were extracted as presented in the original publications, together with the corresponding phenotype scale where available. Because these estimates were not directly comparable across all studies, effect sizes were not quantitatively pooled, standardized, or ranked across heterogeneous analytical scales. Variability in GWAS models and analytical strategies, including adjustment for population structure, relatedness, and covariates, as well as differences in signal selection (e.g., conditional analysis, clumping, or replication frameworks), were summarized and interpreted qualitatively. In addition, similarities and differences in covariate adjustment were considered, as most studies adjusted for age and sex, with the majority also accounting for ancestry or population structure and several including body mass index (BMI) as a covariate. The results were therefore compared across studies in a structured manner, with particular attention to genome-wide significance, replication across independent GWAS, ancestry group of discovery, genomic clustering, ancestry-specific findings and linkage disequilibrium patterns, rather than quantitatively pooled.

### 2.7. Definition of Genomic Clusters for Linkage Disequilibrium Analysis

Given that different GWAS frequently report different lead variants within the same genomic region, often influenced by ancestry, genotyping platform, imputation reference, and study design, an LD-informed framework was applied to distinguish potentially independent signals from correlated proxy variants in a descriptive cross-study framework.

All variants reported as significantly associated with 25OHD levels across published GWAS were first mapped to their genomic coordinates on the GRCh38 reference assembly. To group variants into non-redundant loci, a physical proximity approach was applied. A symmetric window of ±500 kb was constructed around the GRCh38 position of each SNP (start = position − 500,000 bp; end = position + 500,000 bp). This window was selected as a pragmatic and conservative proximity-based locus-definition approach. This window size has been previously used in published GWAS and other genetic association studies [40,41,42], where loci are often defined using the lead variant and flanking regions of approximately 500 kb on either side [40,41,42]. This ±500 kb window is not intended to define LD blocks or to imply that all variants within a cluster represent the same underlying association signal. The specific window was selected to reduce the likelihood that alternative lead variants reported by different GWAS within the same broader regional association signal would be split into separate loci, particularly given that the included studies differed in ancestry composition, genotyping and imputation platforms, phenotype transformation, and signal-selection procedures.

Overlapping windows were iteratively merged to define contiguous genomic regions (clusters). Consequently, clusters could contain one or more variants and were not constrained to a fixed size; their final boundaries were determined by the minimum start and maximum end positions of all contributing SNP windows.

### 2.8. LD Assessment Across Studies

Within each clustering window describe above, pairwise LD was assessed using reference genotype data from the 1000 Genomes Project. The European-ancestry reference panel was used as the primary LD reference, because the majority of reported 25OHD-associated variants were identified in European-ancestry GWAS and because the largest contributing studies were European-ancestry (UK Biobank or SUNLIGHT Consortium) analyses. Where ancestry-matched LD estimates were available for non-European signals, these were considered descriptively; however, the analysis was not designed as a comprehensive ancestry-specific LD comparison across all populations. Pairwise LD (measured as r^2^) was calculated exclusively among the reported GWAS variants within each locus, rather than across all regional variants. Variants in high LD (r^2^ ≥ 0.8) were considered to tag the same underlying genetic signal, whereas variants in low LD (r^2^ < 0.2) were considered potentially independent signals within the same locus. Regions containing only a single reported variant were treated as isolated signals for which LD assessment was not applicable. Because clustering was based solely on physical proximity, the resulting loci could span intergenic regions or multiple genes. Given known differences in allele frequencies and LD structure between populations, LD estimates derived from European reference data may not fully represent correlation patterns in African, Middle Eastern, East Asian, South Asian, Hispanic/Latino, or admixed populations. Therefore, LD-based grouping was interpreted as a descriptive approximation of redundancy among reported variants, rather than as definitive evidence of ancestry-specific signal independence.

Classical LD clumping based on association strength was not applied, as the reported variants originated from heterogeneous GWAS differing in ancestry, phenotype scaling, covariate adjustment, and analytical approach. Instead, LD was used descriptively to evaluate redundancy among reported associations and to estimate the number of LD-distinct signals per locus within the constraints of the available reference data.

## 3. Results

### 3.1. Methodological Characteristics of Included GWAS

Our systematic search of the literature identified 15 genome-wide association studies (GWAS) investigating circulating 25-hydroxyvitamin D (25OHD) concentrations. A total of 349 SNPs with genome-wide statistically significant (*p* < 5 × 10^−8^) associations with 25OHD were identified across the 15 included GWAS. These 349 SNP associations corresponded to 294 unique variants after removal of duplicate reports across studies. Of these, 283 variants were indexed in dbSNP and were therefore retained for subsequent genomic clustering and linkage disequilibrium analyses.

The largest contributions originated from Manousaki et al. [43], who reported 138 SNP associations (39.5% of all SNP–study pairs), and Revez et al. [44], who reported 134 associations (38.4%) [45,46]. Together, these two UK Biobank-based studies accounted for the majority of reported associations, indicating that the current 25OHD GWAS evidence base is strongly shaped by very large European-ancestry biobank analyses. Earlier SUNLIGHT Consortium studies and smaller ancestry-specific GWAS contributed fewer variants, but these remain important for evaluating replication, ancestry representation, and cross-population consistency.

Table 1 summarizes the methodological characteristics of all identified GWAS with circulating 25OHD as an outcome. The main methodological pattern was the marked heterogeneity in the sample size, ancestry composition, phenotype transformation, analytical model, and reporting format, which limited formal quantitative synthesis and motivated a structured qualitative comparison.

### 3.2. Sample Size and Ancestry Profile

There was a very large variation in sample size, ranging from 697 participants in the AA-DHS cohort [45] up to over 400,000 participants in the largest studies primarily leveraging data from the UK Biobank [43,44]. The largest studies were predominantly European-ancestry GWAS, whereas smaller studies contributed evidence from South Asian, African American, Middle Eastern, Hispanic/Latino, and trans-ethnic populations. Thus, although the included literature spans several ancestry groups, the statistical power and variant-discovery yield remain heavily weighted toward European-ancestry datasets.

### 3.3. 25OHD Assessment and Transformation for Analysis

Across the included GWAS, serum 25OHD levels were analysed using heterogeneous outcome scales and statistical summaries, limiting the direct comparison of effect-size magnitudes across studies. Several studies applied the natural log transformation of 25OHD [46], reporting β coefficients that quantify allele-specific changes on the log scale. Other studies [46] reported exponentiated β (multiplicative) rather than absolute changes in 25OHD, while Ahn et al. [47] adopted a square-root transformation. Such studies provide β coefficients that are not directly comparable with those from log-transformed or raw-scale analyses.

In contrast, several large contemporary GWAS, most notably Revez et al. [44], Manousaki et al. [43], Wang et al. (2023) [48], and Hendi et al. [39], applied rank-based inverse-normal transformation (RINT) or explicit internal standardization of 25OHD. In these studies, β estimates represent effects on a transformed or internally standardized phenotype scale. Other analyses modeled 25OHD in raw units or categorical outcomes. Therefore, the effect estimates were interpreted within the scale used by each original study and were not treated as directly comparable across all GWAS.

**Table 1:** Characteristics of 25OHD GWAS included in the systematic review.

Reference	Cohorts/Studies Included	N Sample/SNPs Tested	Ancestry (Specific Population)	Genotype Platform	GWAS Model/Software	25OHD_Scale	Estimate	Relatedness/Structure Control	Signal Selection Method	Covariates	25OHD Variants Reported (n)
Wang et al., 2010 [49]	15 cohorts (5 discovery + 10 replication-SUNLIGHT)	16,125/425,593	European ancestry (British, Swedish, Finnish, Dutch, American)	Multiple cohort-specific SNP arrays	Linear/LMM (MERLIN) → METAL	ln(25OHD)	*p* + effect direction	Genomic control; LMM in related cohorts	Discovery *p* < 5 × 10^−8^ → in silico replication + candidate gene follow-up	Age, sex, BMI, season, site	3
Ahn et al., 2010 [47]	5 primary GWAS: ATBC, CGEMS (PLCO), CPS-II, CLUE II, NHS. Pooled analysis 4 cohorts (ATBC, PLCO, CPS-II, CLUE II)	4501/593,253	European ancestry (Finnish, American)	Illumina Human 550 K (or higher) for ATBC, CGEMS, CPS-II, CLUE II, PLCO, NHS-CGEMS; Affymetrix 6.0 for NHS	Additive linear regression; pooled cohort analysis + √N–weighted Z meta-analysis	sqrt(25OHD)	β	3 PCs; adjustment for study indicator	Signed Wald Z-statistics, meta-analysed using √N weighting; random-effects meta-analysis	Age, sex, BMI, 25OHD batch, study, case-control status, season, vit D suppl, dietary vit latitude, 3 PCs	2
Anderson et al., 2014 [46]	Single cohort	1813/535,632 (imputed to 2,461,244)	European ancestry (Australian)	Illumina Human660W-Quad	Linear regression (ProbABEL)	ln(25OHD)	exponentiated β (multiplicative)	PCA (5 PCs)	GWS threshold only	Age, sex, BMI, season-adjusted vitamin D	3
Sapkota et al., 2016 [50]	Discovery + replication (AIDHS/SDS)	3538/5,904,251	South Asian (Punjabi Indian)	Illumina Human660W-Quad → IMPUTE2 (1000 G)	SNPTEST (Discovery) + SVS LMM (Replication) → METAL	ln(25OHD)	β	5 PCs + IBS filtering	Two-stage *p* < 1 × 10^−4^ → replication	Age, sex, BMI, T2D status	2
O’Brien et al., 2018 [51]	Sub-cohort + replication	3363/386,449	Trans-ethnic (European ancestry primarily, minority with African and Hispanic ancestry)	Illumina OncoArray	Additive linear regression	Raw	β	Ancestry proportions (CEU/YRI/CHB)	*p* < 5 × 10^−8^ threshold with conditional and haplotype analyses	Age, ancestry, sun exposure+ supplementation (sensitivity)	8
Hong et al., 2018 [52]	12 cohorts AA + Hispanic (TRANSCEN-D) + SUNLIGHT	8541 (African American), 3485 (Hispanic), 16,124 (European)/398,246–15,000,000 (depending on cohort)	Trans-ethnic (Hispanic American, African American, European)	Multiple arrays → IMPUTE/MaCH (1000 G)	Linear + LMM (GEMMA/SOLAR) → METAL Z-score	ln(25OHD)	z-score	PC adjustment; mixed models (GEMMA/SOLAR) in family cohorts	*p* < 5 × 10^−8^ Z-score meta	Age, sex, BMI, UV index, PCs	14
Jiang et al., 2018 [53]	Multi-cohort (SUNLIGHT) meta-GWAS	79,366/2,543,887	European ancestry British, Irish, Scottish, Swedish, Finnish, German, Dutch, Italian, American, Canadian)	SNP-arrays (mixed)	Linear regression → METAL meta-analysis	ln(25OHD)	β	PC adjustment; cohort-level stratification control	GWS *p* < 5 × 10^−8^ (QC filtering)	Age, sex, BMI, month (12), PCs, site/batch	4
Revez et al., 2020 [44]	UK Biobank	417,580/8,806,780	European (mainly British), UK	UKB SNP-array → HRC/UK10K imputed	fastGWA LMM (*GC*TA)	RINT	β	Sparse GRM + 40 PCs	COJO + PLINK clumping	Age, sex, month, center, supplements, batch, PCs	134
Manousaki et al., 2020 [43]	UK Biobank	443,734/16,668,957	European (white British), UK	UKB SNP-array → HRC + UK10K	BOLT-LMM	Standardized ln(25OHD)	β (SD-units)	GRM + PCA-defined white British	COJO	Age, sex, season, supplements, batch/array, center	138
Palmer et al., 2021 [45]	AA-DHS cohort	697/1,705,970	African ancestry (African American)	WGS	GEMMA LMM	ln(25OHD + 1)	β	Local + global ancestry (LAMP-ANC/HAPMIX)	*p* < 5 × 10^−8^ + conditional follow-up	Age, sex, BMI, eGFR, supplements, ancestry	6
Kim et al., 2021 [54]	Korea Biobank	7590/1,695,891	East Asian (Korean), South Korea	KoreanChip + Axiom → TOPMed/HRC imputed	Linear + logistic regression	Raw	β (mega-analysis)	PCA (10 PCs), IBS filtering	Mega-analysis inference; LD assessed post hoc	Age, sex, season, supplements, kidney/liver status	3
Parlato et al., 2023 [55]	UK Biobank (AFR)	6934/11,947,647	African or Caribbean descent (British African, British Caribbean), UK	SNP-array	Additive linear regression	ln(25OHD)	β	20 PCs (LD-pruned)	Stepwise ±125 kb + joint conditional	Age, sex, BMI, 20 PCs, genotyping	1
Wang et al., 2022 [13]	UK Biobank (EUR, AFR, SAS, EAS)	8306 (African) 9983 (South Asian), 417,580 (European)/8,546,068–50,357,912	Trans-ethnic (white British, British Indian/Pakistani/Bangladeshi, British African/Caribbean)	UKB Axiom arrays → HRC + UK10K	fastGWA (*GC*TA LMM)	RINT	β	Sparse GRM + 10 PCs	COJO (10 Mb, r^2^ < 0.9)	Age, sex, month, supplements, 10 PCs	4
Hendi et al., 2023 [56]	Qatar Biobank	6047/7,880,618	Middle Eastern (Qatari)	WGS	GRAMMAR-Gamma LMM	RINT	β	Kinship matrix + 4 PCs	LD-clumping (r^2^ < 0.2)	Age, sex, PCs 1–4	1
Hendi et al., 2025 [39]	Qatar Biobank	13,652/49,260,795–56,600,172	Middle Eastern (Qatari)	WGS	SAIGE LMM → PLINK meta-analysis (fixed-effects inverse-variance weighting)	RINT	β	SAIGE mixed model (sparse GRM) + 4 PCs for structure	Two-stage rare-variant design; ±250 kb regional follow-up; fixed-effects IVW meta-analysis	Age, sex, PCs 1–4	6

METAL: Fast and efficient meta-analysis of genome-wide association scans tool; LMM: linear mixed model; RINT: rank-based inverse-normal transformation; PCs: principal components; GRM: genomic relationship matrix; COJO: conditional and joint analysis; LD: linkage disequilibrium.

### 3.4. GWAS Models and Analysis

GWAS models ranged from additive linear regression to linear mixed models, with multi-cohort studies commonly using meta-analytic frameworks. Most studies adjusted for core covariates such as age, sex, ancestry or population structure and study site or batch, while the adjustment for BMI, season, and vitamin D supplementation varied. Differences in model specification, relatedness adjustment, covariate selection, and signal-selection procedures, including conditional analysis or LD clumping, were therefore considered qualitatively rather than used for formal cross-study effect-size comparison.

### 3.5. Reporting of Main Effects

Several studies reported test statistics rather than effect sizes. Hong et al. [52] provided only Z-scores from METAL, reflecting directional significance rather than magnitude of effect, which limited interpretation of effect magnitude. Similarly, Wang et al. (2010) [49] reported *p*-values and effect directions only, precluding direct comparison of effect sizes with studies reporting β coefficients and standard errors. Kim et al. [54] performed both mega- and meta-analyses, with the mega-analysis providing effect estimates (β coefficients) and the meta-analysis yielding z-scores.

Overall, the studies varied substantially in transformation choices and reporting formats. For this reason, the reported effects were extracted as presented in the original publications and interpreted in relation to their original analytical scale. Cross-study synthesis therefore focused on consistently comparable features, including genome-wide significant loci, replication across independent GWAS, ancestry group of discovery, genomic clustering, and LD structure.

### 3.6. Methodological Quality of Studies

Supplementary Table S2 summarizes the methodological quality and reported limitations of the included GWAS. Our formal evaluation of study power revealed that the power adequacy differed markedly across the included GWAS and ancestry-specific analysis strata. Fourteen of the 15 included studies met the predefined power threshold under at least one allele-frequency/effect-size scenario, whereas Palmer et al. did not meet the threshold under any assessed scenario. The largest GWAS, particularly Revez et al. [44]. and Manousaki et al. [43] showed the broadest power adequacy, including for weak-effect high-frequency variants and borderline power for weak-effect low-frequency variants. Jiang et al. [53] also showed relatively broad power adequacy, although not for weak-effect scenarios. In contrast, smaller studies and several non-European ancestry-specific analyses were generally powered only for strong-effect variants.

Methodological heterogeneity was also evident, including differences in genotyping platforms, imputation approaches, and phenotype measurement, particularly with respect to assay variability. In addition, incomplete adjustment for environmental and lifestyle factors, such as diet, supplementation, and seasonality, was frequently reported. Several studies also noted challenges related to causal inference, including uncertainty in identifying functional variants and the lack of functional validation. Finally, potential sources of bias, including cohort selection bias and demographic imbalances, were reported across a number of studies.

### 3.7. GWAS-Identified 25OHD Variant Characteristics Across Studies

Across the 15 GWAS included in our review, we identified 294 unique genetic variants (after removing duplicates and triplicates) associated with 25OHD levels (Supplementary Table S3). The largest contributions of unique variants originated from the UK Biobank-based GWAS by Revez et al. [44] and Manousaki et al. [43], accounting for 45.4% and 36.3% of identified variants, respectively (Table 1). This concentration confirms that most variant discovery in the current literature is driven by a small number of very large European-ancestry GWAS.

**Figure 2:**
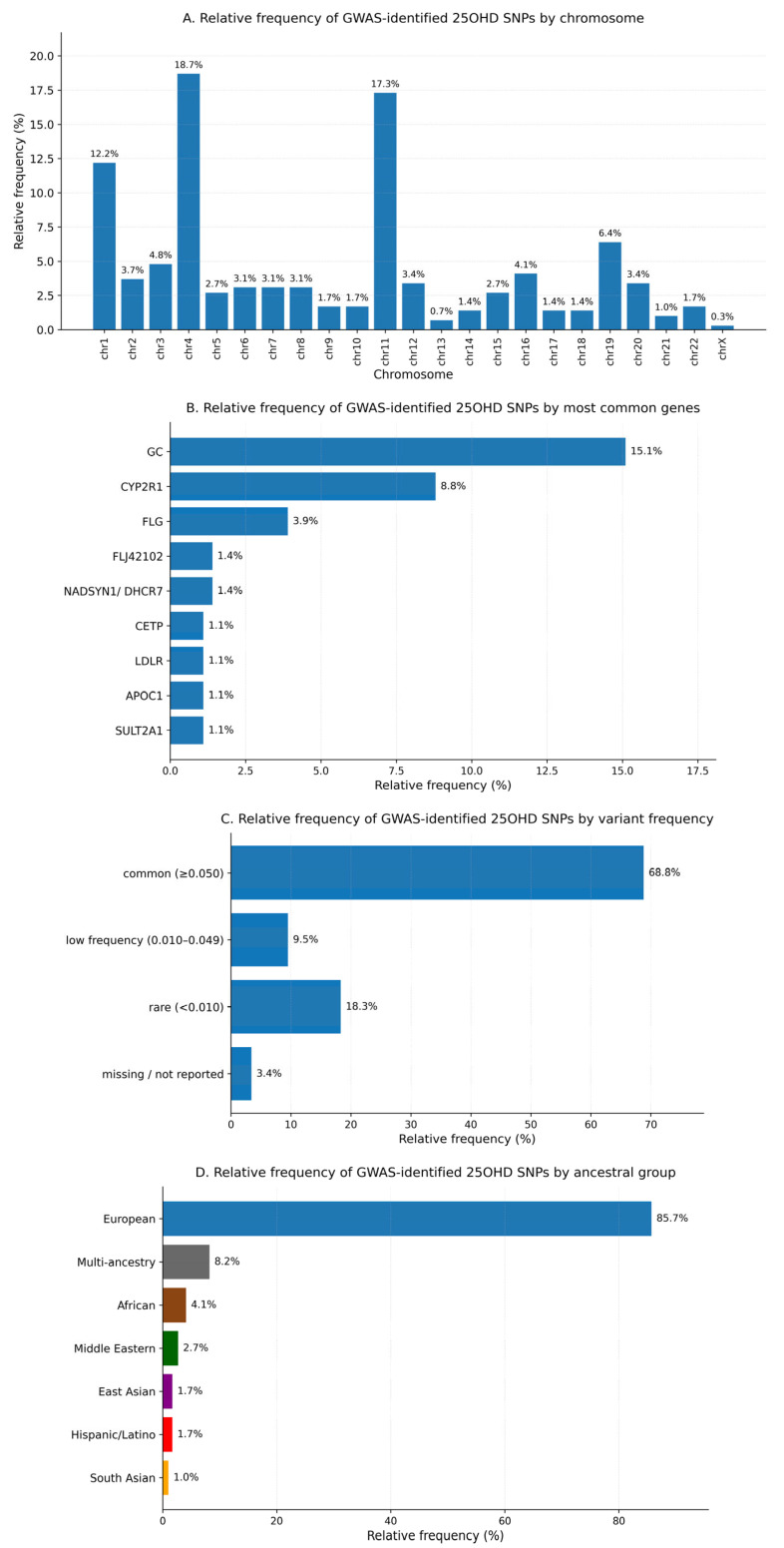
Overview of GWAS-identified 25OHD-associated SNPs. Percentages are calculated using the 283 dbSNP-indexed variants retained for linkage disequilibrium analyses unless otherwise stated. The total number of unique genome-wide significant variants identified across all included GWAS was 294.

Figure 2 provides an overview of GWAS-identified 25OHD-associated SNPs. For clarity, percentages reported throughout the Results section are calculated using the relevant denominator, including genome-wide significant SNP associations (n = 349), unique variants (n = 294), dbSNP-indexed variants retained for LD analyses (n = 283), or gene-mapped variants where applicable. Of these, 283 variants were indexed in dbSNP and were retained for all downstream genomic and linkage disequilibrium (LD) analyses.

The 283 dbSNP-indexed variants were distributed across multiple chromosomes. The highest concentrations of SNPs were observed on chromosome 4 (55 SNPs; 19.4%) and chromosome 11 (52 SNPs; 18.7%), followed by chromosome 1 (36 SNPs; 12.3%), chromosome 19 (19 SNPs; 6.7%), and chromosome 2 (14 SNPs; 4.9%). The remaining chromosomes each harbored comparatively smaller numbers of associated variants. This distribution indicates that 25OHD-associated signals are genome-wide but are disproportionately concentrated in a limited number of chromosomal regions.

Most variants were common (202/294 unique variants, 68.8%), while 28/294 (9.5%) were low-frequency, and 54/294 (18.3%) were rare. Allele frequency information was missing for 10/294 variants (3.4%), which were not indexed in dbSNP. The predominance of common variants reflects the design and power profile of most included GWAS, whereas rare and low-frequency findings were less frequent and more dependent on study-specific discovery settings.

Overall, the majority of variants were identified in populations of European ancestry (252/294 unique variants, 85.7%), with substantially fewer variants reported in populations with other ancestral backgrounds, namely African (12 SNPs, 4.1%), Middle Eastern (8 SNPs, 2.7%), East Asian (5 SNPs, 1.7%), Hispanic/Latino (5 SNPs, 1.7%), and South Asian (3 SNPs, 1.0%). A number of SNPs (n = 24, 8.2%) were identified in trans-ethnic/multi-ancestry analyses, usually comprising individuals from European, African, and Hispanic ancestral backgrounds [51,52]. Thus, the observed ancestry distribution of reported variants should be interpreted primarily as a reflection of study availability and statistical power, rather than as definitive evidence that 25OHD genetic architecture is predominantly European-specific.

Overall, the 283 variants mapped to 170 unique genes (Supplementary Table S4). Figure 2 lists the genes with the highest number of 25OHD-associated variants.

The most extensive aggregation of SNPs was observed at the *GC* locus (43 SNPs; 15.1%), followed by *CYP2R1* (25 SNPs; 8.8%) and *FLG* (11 SNPs; 3.9%. A smaller number of variants were mapped to *FLJ42102* (4 SNPs; 1.4%), as well as the *NADSYN1*/*DHCR7* locus (4 SNPs; 1.4%), while several additional genes, including *CETP*, *LDLR*, *APOC1*, *CYP24A1*, and *SULT2A1*, each harbored three associated SNPs (1.1%). Most remaining genes contained one–two associated variants.

These findings indicate that, although 25OHD-associated variants are distributed across the genome, their aggregation is disproportionately concentrated within a limited number of biologically relevant loci.

### 3.8. Strength of Genetic Associations with Circulating 25OHD

The median *p*-value was 1.35 × 10^−12^ (interquartile range: 4.64 × 10^−26^ to 1.82 × 10^−9^; range: 3.9 × 10^−295^ to 5.31 × 10^−8^), demonstrating strong statistical evidence across reported associations. However, the statistical significance was strongly influenced by the study sample size, particularly in the largest UK Biobank-based GWAS, and should not be interpreted as directly proportional to the biological effect magnitude. Table 2 presents the GWAS-identified SNPs with the smallest *p*-values in their association with 25OHD levels, across studies.

As regards effect estimates, caution should be taken as regards direct comparisons of study-reported effect sizes, as these rely heavily on the transformation of the outcome variable (numeric 25OHD concentrations) in the original analysis (Supplementary Table S1). Because the included GWAS used raw, log-transformed, square-root-transformed, rank-based inverse-normal transformed, standardized, or categorical/binary outcomes, the reported effect estimates were not standardized, harmonized, quantitatively pooled, or ranked across studies. Instead, the effect estimates are reported in Supplementary Table S3 as presented in the original publications, together with the relevant phenotype scale where available. The most statistically significant associations were concentrated primarily at established vitamin D-related loci, particularly GC and CYP2R1, supporting the central role of these loci in the genetic architecture of circulating 25OHD.

### 3.9. Replication Across Studies

**Figure 3:**
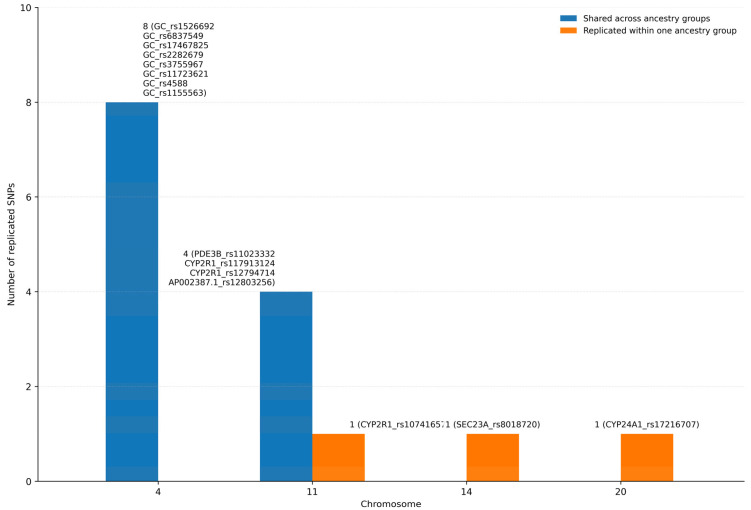
Replicated 25OHD-associated SNPs by chromosome and ancestry-specific sharing. Repeated evidence was defined conservatively by grouping substantially overlapping study resources together. Revez et al. [44]. and Manousaki et al. [43] were treated as one UK Biobank-based study group, and Wang et al. (2010) [49] and Jiang et al. [53] were treated as one SUNLIGHT-based study group. Variants repeated within these overlapping groups were not considered evidence of independent replication. Detailed information about cross-GWAS replicated SNPs can be found in Supplementary Table S5.

A subset of variants demonstrated reproducibility across independent GWAS. Figure 3 summarizes the replication consistency across independent cohorts by detailing the number of contributing studies for each variant and the corresponding ancestral backgrounds in which the associations were identified.

Of the 294 unique variants identified across all included studies, 15 (5.1%) were reported in at least two independent GWAS. Three SNPs (20% of replicated variants) were identified in three independent GWAS each: rs11723621, rs4588, and rs2282679 within the *GC* locus. The remaining 12 replicated variants (80%) were reported in two independent cohorts. The replicated variants were concentrated mainly at established vitamin D loci, especially *GC* and *CYP2R1*, with additional replicated signals at or near *SEC23A*, CYP24A1, AP002387.1, and PDE3B. This indicates that only a small proportion of reported variants have recurred across independent GWAS, and replication is strongest for well-established biological loci.

### 3.10. Replication Across Different Ancestries and Identification of Ancestry-Specific Variants

Cross-ancestry replication was observed for 15 replicated variants. Three variants were identified across three distinct ancestral backgrounds. SNP rs11723621 in *GC* was identified in European, Middle Eastern, and East Asian populations [43,54,56], while rs4588 in *GC* was reported in Middle Eastern and African populations, as well as in a trans-ethnic analysis [51,55,56]. Similarly, rs2282679 in *GC* was observed in European populations and trans-ethnic analyses [47,49,52].

Twelve additional variants demonstrated replication across two ancestral backgrounds. rs12803256 (*AP002387.1*) and rs11023332 (*PDE3B*) were identified in European and East Asian populations [43,44,46,54], whereas rs1352846 (*GC*) was replicated in European and African ancestry groups [44,48]. rs12794714 and rs117913124 (*CYP2R1*) were identified in European and trans-ethnic populations [44,51], while rs3755967 (*GC*) was observed in European and Hispanic ancestry groups [52,53], and rs17467825 (*GC*) was replicated in European and South Asian populations [46,48]. In addition, three variants in high linkage disequilibrium with rs4588, namely rs1155563, rs1526692, and rs6837549 (*GC*), were also observed across more than one ancestral background, involving combinations of European, Hispanic, and trans-ethnic analyses [46,51,52].

It should be noted that trans-ethnic GWAS often combine participants from multiple ancestral backgrounds to increase the statistical power and variant discovery. While such designs provide valuable insights into shared genetic architecture, they may not offer the same level of ancestry-specific resolution as analyses conducted within individual ancestry groups and may therefore mask population-specific genetic effects.

Ancestry-specific genetic signals among non-European populations can be found in Table 3. The table presents genomewide-significant SNPs observed only in specific ancestral groups, as denoted below. This table is complemented by Supplementary Table S8, which additionally includes all SNPs identified in a specific ancestral group, including both ancestry-specific SNPs and SNPs replicated in other ancestral groups.

In individuals of African ancestry, a relatively large number of SNPs were reported primarily mapping to loci other than the major ones (e.g., *HSPG2*, *TNIK*). In Hispanic populations, fewer variants were identified primarily within the *GC* locus. Middle Eastern populations demonstrated several ancestry-specific variants spanning different loci. In South Asian populations, the identified ancestry-specific variants were restricted to a single locus mapping to the *FOXA2/SSTR4* region. Similarly, in East Asian populations, ancestry-specific variants were observed in two major loci (*GC*, and *NADSYN1*).

### 3.11. LD-Based Clustering of 25OHD-Associated Variants

To determine whether genetic variants associated with circulating 25OHD levels and reported across independent GWAS represent distinct underlying genetic signals or reflect redundant associations, LD was investigated among reported GWAS variants within proximity-defined genomic clusters.

For each of the 283 unique dbSNP-indexed variants identified from eligible GWAS, genomic coordinates were harmonized to the GRCh38 reference build. Variants were then grouped into preliminary loci based on physical proximity by defining ±500 kb windows around each variant and merging overlapping intervals within each chromosome. These proximity-based loci (clusters) were used solely as an organizational framework and did not assume an a priori LD structure. This procedure yielded 128 distinct genomic clusters comprising the 283 reported variants. Of these clusters, 83 contained a single 25OHD-related variant (excluded from downstream LD analysis and not presentment in Supplementary Table S11), 29 clusters contained two variants, and 16 clusters contained three or more variants. Thus, most loci contained only one reported variant, while multi-variant clusters represented a smaller subset of regions where LD assessment was informative for distinguishing correlated from potentially independent reported signals.

Detailed cluster counts and high-LD groups are provided in Supplementary Table S11. In the main Results, the most informative pattern was that multi-SNP clusters were concentrated within a limited number of genomic regions, while most clusters were small and contained few reported variants.

The largest multi-SNP clusters were concentrated within a limited number of genomic regions. Cluster 98 on chromosome 4 contained the highest number of variants (44 SNPs) and spanned the *GC* and RP11-545L5.1 genes. This was followed by cluster 20 on chromosome 11, which included 30 SNPs mapped to genes *CYP2R1*, *PDE3B*, and PSMA1, and cluster 11 on chromosome 1, comprising 15 SNPs spanning genes *FLG*, *FLG*-*AS1*, *RP11-107M16.2*, and *HDHD1P2*. Cluster 23 on chromosome 11 contained 13 SNPs within the *DHCR7*/*NADSYN1* genetic region. These clusters correspond to biologically plausible vitamin D-related regions and account for much of the apparent recurrence of reported 25OHD-associated variants across studies.

### 3.12. Pairwise LD Structure Within Clusters

LD analyses were performed within clusters containing ≥2 variants using 1000 Genomes Project. Pairwise LD was evaluated within multi-variant clusters using European-ancestry reference data. LD estimates were available for 961 of 967 within-cluster variant pairs. Supplementary Table S11 displays LD groups within clusters and number of high LD pairs (r^2^ > 0.80) within groups.

Among non-missing pairs, 66/961 (6.9%) exhibited high LD (r^2^ ≥ 0.80), 176/961 (18.3%) showed moderate LD (0.20 ≤ r^2^ < 0.80), and 719/961 (74.8%) demonstrated low LD (r^2^ < 0.20), indicating that most within-cluster variant pairs were weakly correlated. This suggests that physical proximity alone often grouped variants that were not strongly correlated and may represent distinct reported signals within broader genomic regions.

High-LD variant pairs were concentrated in a limited number of clusters. The highest density was observed in cluster 98, which contained 29 high-LD pairs and spanned the *GC* and RP11-545L5.1 loci. Clusters 20 and 23 each contained eight high-LD pairs and encompassed genes including *CYP2R1*, *PDE3B*, PSMA1, *DHCR7*, and *NADSYN1*. Smaller numbers of high-LD pairs were observed in cluster 63 (two pairs; *APOC1* and APOE) and cluster 1 (one pair; PEX10 and RER1). Therefore, LD redundancy was not distributed uniformly across the genome but was concentrated in a small number of established 25OHD-associated regions.

Overall, the LD analysis identified a limited number of high-LD groups within seven multi-variant clusters, primarily involving GC, CYP2R1/PDE3B/PSMA1, DHCR7/NADSYN1, and APOC1/APOE regions. Qualitative inspection of LD patterns revealed substantial heterogeneity across clusters. Some clusters, such as cluster 98, exhibited extensive high-LD structure with multiple overlapping LD blocks, indicating redundancy driven by dense local LD. In contrast, other clusters (e.g., cluster 11) showed predominantly low LD, suggesting the presence of largely independent association signals within the same genomic region. Several smaller clusters, including clusters 41, 60, and 61, demonstrated near-complete LD, consistent with single underlying association signals. Clusters such as 20 and 23 displayed more complex LD architectures, with multiple high-LD subgroups interspersed with regions of lower LD, suggesting the presence of correlated but non-uniform signals within these loci.

For each LD group, we assigned a lead SNP, based on (a) strength of association (lowest 25OHD association *p*-value) and (b) replication across GWAS. In some high-LD pairs, alternative variants showed similar association strength or replication consistency; in these cases, lead-SNP assignment should be interpreted pragmatically rather than as definitive evidence of causality.

## 4. Discussion

This systematic review identified 294 unique genetic variants associated with circulating 25-hydroxyvitamin D concentrations across 15 GWAS [39,43,44,45,46,47,48,49,50,51,52,53,54,55,56]. The findings support a predominantly polygenic genetic architecture, with the strongest and most consistently reported signals mapping to biologically established vitamin D-related loci, particularly GC, CYP2R1, and DHCR7/NADSYN1.

### 4.1. Overall Genetic Architecture of Circulating 25OHD

Across the 15 included GWAS, 349 genome-wide significant SNPs were reported, corresponding to 294 unique variants. Of these, 283 variants were indexed in dbSNP and retained for downstream genomic and LD analyses. LD-based clustering under a ±500 kb proximity framework yielded 128 distinct genomic loci. Most loci were small, with 83 containing a single variant, 29 containing two variants, and 16 comprising three or more variants. The majority of variants were detected in the *GC* gene, followed by *CYP2R1*. Other genes harboring a significant number of 25OHD variants were *NADSYN1*/*DHCR7*, *FLG*, and *FLJ42102*.

Within multi-variant clusters, nearly three-quarters of pairwise variant comparisons demonstrated low LD (r^2^ < 0.20), while only 6.9% exhibited high LD (r^2^ ≥ 0.80). This distribution indicates that the majority of reported associations are not simply reflections of dense local correlation structures. Although certain genomic regions exhibited concentrated clustering, the overall distribution of variants was broad across the genome.

Study-level replication was present but limited relative to the total number of identified variants. Of the 294 unique variants identified across all included studies, 15 (5.1%) were reported in at least two independent GWAS. Three SNPs were identified in three independent GWAS each, while the remaining 12 replicated variants were reported in two independent studies.

These replicated SNPs mapped predominantly to established vitamin D-related loci, including *GC*, *CYP2R1*, and *CYP24A1*, but also extended to additional genomic regions such as *AP002387.1*, *ZNF259*, *POU2F3*, *ALDH1A2*, *PEAK1*, *PDILT*, *DSG1*, *SERPINB11*, *NPHS1*, *KLK10*, *BCAS1*, and *PDE3B*.

Overall, these findings support a predominantly polygenic architecture for circulating 25OHD concentrations. While specific loci harbor dense aggregations of associated variants, most variants appear to represent independent or weakly correlated genomic signals.

### 4.2. Strength of Associations, LD Structure and Independence of Signals

Variants with the smallest *p*-values were predominantly located at *GC* and *CYP2R1*, confirming their dominant contribution to statistical signal strength.

Substantial methodological heterogeneity was observed across studies in transformation and reporting of 25OHD outcomes, including log-transformed, square-root–transformed, raw-scale, and rank-based inverse-normal transformed analyses.

The LD-based clustering framework provided insight into the structural independence of reported associations. Among 961 evaluable within-cluster variant pairs, 66 (6.9%) exhibited high LD (r^2^ ≥ 0.80), 176 (18.3%) demonstrated moderate LD, and the majority (74.8%) showed low LD. These findings indicate that redundancy due to dense LD is concentrated within specific genomic regions rather than being a pervasive feature of the vitamin D genomic landscape.

Clusters such as the *GC* locus displayed an extensive high-LD structure with multiple correlated variants, whereas other clusters showed predominantly low LD, consistent with largely independent association signals within shared genomic windows.

These distinctions are particularly relevant for downstream applications such as Mendelian randomization and polygenic risk modeling, where assumptions regarding instrument independence and LD pruning are critical.

This review provides a systematic and structured synthesis of all GWAS-identified variants for circulating 25OHD, integrating study-level replication with LD-based locus clustering. By examining SNP-level, locus-level, gene-level, and LD-group structure in parallel, the analysis offers a multi-layered characterization of the genetic architecture underlying vitamin D status. Several limitations warrant consideration. First, LD analyses were conducted using European-ancestry reference panels, which may not fully capture LD structure in non-European populations. Second, the ±500 kb clustering framework, while methodologically consistent, does not account for long-range regulatory interactions. Third, replication was assessed descriptively based on reported GWAS findings rather than through harmonized re-meta-analysis of effect estimates. Finally, functional annotation and mechanistic validation were beyond the scope of this review.

### 4.3. Locus-Level Concentration and Biological Relevance

Despite the broad genomic distribution, the variant aggregation was disproportionately concentrated at biologically established vitamin D-related loci. The strongest clustering was observed at the *GC* locus, encoding the vitamin D-binding protein, followed by *CYP2R1*, encoding the principal hepatic 25-hydroxylase.

Cluster 98 on chromosome 4 (*GC* region) contained the largest number of variants (44 SNPs), followed by cluster 20 on chromosome 11 (*CYP2R1*/*PDE3B*/PSMA1 region) with 30 variants, cluster 11 on chromosome 1 (15 variants spanning *FLG* and related genes), and cluster 23 within the *DHCR7*/*NADSYN1* region (13 variants). The concentration of variants within these biologically central pathways is in concordance with their established role in vitamin D physiology and homeostasis and, in particular, vitamin D transport and metabolism.

The *GC* gene encodes the vitamin D-binding protein (DBP), the principal carrier of circulating 25OHD, and genetic variation at this locus has been consistently associated with altered total 25OHD concentrations, potentially reflecting differences in binding affinity, circulating DBP levels, and bioavailable vitamin D fractions [57,58,59,60]. Common missense variants such as rs4588 and rs7041 define the major DBP isoforms, which differ in both serum concentration and ligand-binding affinity, producing predictable shifts in measured 25OHD across genotypes. These allelic differences have been shown to influence not only total 25OHD but also DBP abundance and urinary DBP excretion, reinforcing the idea that *GC* variation modulates vitamin D status through both quantitative and qualitative effects on the carrier protein.

*CYP2R1* is a cytochrome P450 25-hydroxylase that encodes the primary hepatic 25-hydroxylase responsible for converting vitamin D into 25OHD; thus, functional variation in this gene may influence enzymatic efficiency and thereby directly modulate circulating 25OHD levels [49,60,61]. In particular, *CYP2R1* catalyzes the C-25 hydroxylation of both vitamin D_2_ and D_3_, a critical first step in generating 25OHD. This enzymatic role is well established through biochemical studies demonstrating its ability to hydroxylate native vitamin D substrates and several vitamin D analogues [60,61]. Missense or regulatory variants may reduce enzyme activity, lowering the rate of hepatic 25-hydroxylation and directly decreasing circulating 25OHD [62]. In addition, changes in *CYP2R1* expression or function can shift the balance of vitamin D metabolism, affecting how efficiently dietary or cutaneously produced vitamin D is converted into its measurable circulating form [63,64]. Finally, with *CYP2R1* acting upstream of renal 1α-hydroxylation, variation at this locus can influence the availability of substrate for downstream activation to 1,25(OH)_2_D [63,64].

*DHCR7* encodes 7-dehydrocholesterol reductase, the enzyme that converts 7-dehydrocholesterol (7-DHC) to cholesterol and thereby controls the pool of 7-DHC available for UVB-driven cutaneous conversion to previtamin D3 [49,53,64], and common variants at the adjacent *DHCR7*/*NADSYN1* locus (e.g., rs12785878) are among the most consistently replicated genetic determinants of circulating 25OHD, a finding best explained by a substrate-partitioning model in which alleles that reduce *DHCR7* expression or activity increase 7-DHC availability and raise vitamin D synthesis. *NADSYN1* (encoding an NAD^+^ synthetase) lies within the same association interval and could plausibly modify 25OHD indirectly via effects on NAD^+^/NADP(H)-dependent redox and cofactor supply for CYP-mediated vitamin D hydroxylation or via NAD^+^-sensitive signaling in skin, although functional follow-up to date more strongly implicates *DHCR7* as the primary causal gene at this locus [44,49,53].

As regards other involved genes, *FLG* encodes filaggrin, a key structural protein of the epidermal barrier, and loss-of-function variants may influence cutaneous UV penetration and endogenous vitamin D synthesis through effects on skin barrier integrity [65,66]. Although the functional role of *FLJ42102* remains less clearly characterized, its recurrent association with 25OHD concentrations suggests proximity to regulatory elements or linkage with functional variants within relevant metabolic pathways [43,44,53], but such possible mechanistic implications should be interpreted with caution.

Collectively, the convergence of strong statistical signals, locus-level clustering, and biologically coherent gene functions highlights the central role of GWAS-identified variants in the genetic architecture underlying circulating 25OHD concentrations, while acknowledging that functional validation studies are required to establish precise mechanistic pathways.

In particular, post-GWAS functional annotation is needed to distinguish causal genes from nearby correlated variants within 25OHD-associated loci. eQTL and splicing-QTL analyses can help assess whether associated variants influence gene expression or transcript structure in tissues relevant to vitamin D metabolism, including liver, skin, and kidney. Public resources such as GTEx [67], which provides tissue-specific gene-expression and regulatory data across multiple human tissues, and the eQTL Catalogue [68], which provides uniformly processed human expression and splicing QTLs, can potentially support colocalization analyses between 25OHD GWAS signals and molecular traits. However, such discussion is beyond the scope of our review.

### 4.4. Ancestry-Specific Variation

Replication was largely confined to European-ancestry GWAS, consistent with the ancestry distribution of the identified variants (81.6%). However, a subset of variants demonstrated cross-ancestry reproducibility across two or more ancestral backgrounds, including European, African, Middle Eastern, East Asian, Hispanic/Latino, South Asian, and trans-ethnic analyses. This observation raises an important question: do different ancestral groups exhibit distinct genetic architectures for circulating 25OHD concentrations, or do they share a core set of biological determinants?

The evidence synthesized in this review suggests that the underlying 25OHD genetic architecture is largely shared across ancestries (Figure 2). Variants within the *GC* locus (including rs11723621, rs4588, and rs2282679) and *CYP2R1* (including rs10741657), as well as rs12803256 (*AP002387.1*) and rs8018720 (SEC23A), were identified across multiple ancestral groups. These loci encode proteins central to vitamin D transport and metabolic activation, providing strong biological plausibility for their reproducibility across diverse populations. Genes directly involved in vitamin D synthesis, hydroxylation, and circulation would be expected to influence circulating 25OHD concentrations irrespective of continental ancestry, although the magnitude of association may vary due to differences in allele frequency distributions and LD structure.

Importantly, several of these cross-ancestry variants also represent among the most statistically robust associations in the dataset, suggesting that they reflect fundamental determinants of vitamin D physiology rather than population-specific artifacts. Nevertheless, the current review does not include harmonized cross-ancestry effect size comparisons; therefore, it cannot determine whether their quantitative impact differs systematically between populations.

While the majority of replicated variants were confined to European-ancestry GWAS, some replicated variants were consistently observed only among non-European populations. A characteristic example is variant rs4588 in *GC*, which has been observed among African-Caribbean, Middle Eastern, and multi-ancestry cohorts, but was not picked up by the sufficiently powered large European cohorts (e.g., UK Biobank and SUNLIGHT Consortium). This is, therefore, a major non-European 25OHD-associated candidate signal. Although the present review was not designed to test evolutionary selection directly, the repeated identification of GC variants across ancestry groups, together with signals at loci involved in vitamin D metabolism and cutaneous UV-related pathways, is consistent with the broader hypothesis that population differences in 25OHD genetics may reflect both shared metabolic determinants and ancestry-related differences in allele frequencies and LD structure shaped partly by evolutionary UV adaptation.

Several other loci demonstrated population-restricted associations, particularly in African, Middle Eastern, South Asian, and East Asian populations (Table 3 and Table S8). These ancestry-specific signals were observed both within established vitamin D-related loci, such as *GC*, and across a range of additional genomic regions, including *HSPG2*, *TNIK*, *CNTN3*, *EBF1*, *FOXA2/SSTR4*, and *NADSYN1*. It could be speculated that the presence of such population-specific variants suggests a slightly differing genetic architecture of circulating 25OHD concentrations across ancestral groups, which could reflect population-specific allele frequencies, linkage disequilibrium structure, or gene–environment interactions.

Overall, it should be noted that due to the predominance of large European cohorts, especially the UK Biobank, many of the above-described ancestry-specific findings may reflect the unequal distribution of available scientific evidence and study power limitations in non-European GWAS, rather than true biological differences among ancestries.

### 4.5. Data Synthesis Limitations

The present review has some limitations that should be considered when interpreting the findings. First, a formal meta-analysis was not feasible because of the substantial methodological heterogeneity across the included GWAS. Studies differed in the assessment and modeling of circulating 25-hydroxyvitamin D (25OHD), including the use of raw values, log-transformed or square-root–transformed measures, rank-based inverse-normal transformation, standardized continuous phenotypes, and categorical or binary vitamin D outcomes. Additional variability in statistical models, covariate adjustment strategies, ancestry composition, discovery and replication designs, and reporting of effect estimates further limited direct comparability across studies. For this reason, effect estimates were extracted as reported in the original publications but were not standardized, harmonized, quantitatively pooled, or ranked across studies. Consequently, the review does not provide a formal comparison of effect-size magnitudes across GWAS; instead, findings were synthesized using a structured qualitative framework focused on genome-wide significant loci, replication across independent GWAS, ancestry group of discovery, genomic clustering, and linkage disequilibrium patterns.

In addition, the LD estimates were based on European-ancestry reference data from the 1000 Genomes Project, since the vast majority of identified genomewide significant in our review were identified in populations of European descent. Whether this is representative of LD patterns among non-Europeans is questionable, given that the LD structure and allele-frequency distributions may differ in non-European populations.

Furthermore, it should be noted that, although the ±500 kb clustering window used in our review to identify independent signals across studies, has been consistently used in the literature, it is an arbitrary block size, something that could potentially influence the LD estimation between cross-study identified variants. In addition, pairwise LD (measured as r^2^) was calculated exclusively among the reported GWAS variants within each locus, rather than across all regional variants; therefore, this descriptive approach may not capture unreported proxy variants, regulatory variants, or other potentially relevant variants present within the same genomic window but not reported in the original GWAS.

Other limitations pertain to the available GWAS literature itself. Most included studies were conducted in populations of predominantly European ancestry, preventing a robust comparison of genetic variation across ancestral groups. Furthermore, some studies were based on partially overlapping cohorts, particularly large biobank-based and consortium-based datasets. Additional heterogeneity was observed in phenotype transformation procedures, statistical modeling approaches, covariate adjustment strategies, and replication practices. Finally, functional validation remains unavailable for many reported associations, limiting the biological interpretation of the identified genetic signals.

The findings of this review may have potential implications for public health and future precision-nutrition approaches. Although ancestry-related genetic variation may contribute to differences in circulating 25OHD concentrations across populations, these findings should not be interpreted deterministically or independently of environmental influences. Vitamin D status remains strongly affected by factors such as sun exposure, dietary intake, supplementation practices, lifestyle characteristics, and underlying health conditions. Nevertheless, improved understanding of ancestry-specific genetic architecture may contribute to future research aiming to refine risk stratification, vitamin D assessment, and precision supplementation strategies.

At present, however, the available evidence remains insufficient to support ancestry-specific vitamin D supplementation recommendations or population-specific clinical thresholds, and clinical interpretation of serum 25OHD concentrations should continue to rely on established clinical guidelines together with the broader environmental and clinical context.

## 5. Conclusions

This systematic review provides a comprehensive synthesis of the genome-wide association evidence on circulating 25-hydroxyvitamin D concentrations. The marked imbalance in representation across ancestral groups, particularly those of non-European descent, remains a major limitation in the field. Expanded genomic studies in underrepresented populations will therefore be critical to disentangle true ancestry-specific effects from artefacts of study design and study power. Such data, when available, could potentially inform precision public health approaches for tackling disparities in vitamin D deficiency in diverse populations, both within and across countries.

## Figures and Tables

**Table 2:** GWAS-identified SNPs with the smallest *p*-values.

SNP	Gene	*p*-Value *	Effect Size	GWAS Identified (Reference)
rs1352846	*GC*	0	0.193	Revez et al., 2020 [44]
rs12794714	*CYP2R1*	0	0.088	Revez et al., 2020 [44]
rs12798050	*S100A11P3*	0	−0.110	Revez et al., 2020 [44]
rs12803256	*AP002387.1*	0	−0.104	Revez et al., 2020 [44]
rs116970203	*PDE3B*	0	0.377	Revez et al., 2020 [44]
rs11723621	*GC*	2.903 × 10^−1689^	−0.187	Manousaki et al., 2020 [43]
rs117913124	*CYP2R1*	1.653 × 10^−775^	−0.354	Manousaki et al., 2020 [43]
rs3755967	*GC*	4.740 × 10^−343^	−0.216	Jiang et al., 2018 [53]
rs577185477	*CYP2R1*	1.624 × 10^−342^	−0.379	Manousaki et al., 2020 [43]
rs3775150	*GC*	3.900 × 10^−295^	−0.091	Manousaki et al., 2020 [43]

* *p*-values reported as “below reporting threshold” reflect extremely small *p*-values, which were reported as ‘0’ in the original GWAS and were reported as such in the original studies; therefore, there was no way of retrieving the actual value. Thus, zeros indicate extremely small *p*-values and are placed at the top of the list, as regards strength of association with 25OHD.

**Table 3:** GWAS-identified ancestry-specific SNPs among non-Europeans.

Ancestry	Ancestry-Specific SNPs (Gene)
African ancestry	rs116788687 (*HSPG2*) rs143555701 (*TNIK*) rs116950775 (*KIAA1644/LDOC1L*) rs114001906 (*FLJ31813*) rs111955953 (-/*FTMT*) rs117075918 (*TBC1D16*) rs146759773 (*PRKD3*) rs843005 (*GC*) rs222040 (*GC*) rs79666294, (*KIF4B*)
Hispanic ancestry	rs377687 (*GC*) rs56003670 (*GC*)
Middle Eastern ancestry	rs2298850 (*GC*) rs115651661 (*CNTN3*) rs536115678 (*EBF1*) chr21:43954055:C:T (*AGPAT3*) chr21:43790823:A:G (*RRP1*) rs550626115 (*TPM1–AS*) rs1014490316 (*PHACTR3*)
South Asian ancestry	rs6048371 (*FOXA2/SSTR4*) rs2207173 (*FOXA2/SSTR4*)
East Asian ancestry	rs7041 (*GC*) rs3831470 (*NADSYN1*)

## Data Availability

No new data were created or analyzed in this study. Data sharing is not applicable to this article.

## Supplementary Materials

The following supporting information can be downloaded at https://www.mdpi.com/article/10.3390/nu18132052/s1, Table S1. PubMed search algorithm. Table S2. GWAS quality assessment. Table S3. all GWAS SNPs. Table S4. dbSNP indexed. Table S5. SNPs by chromosome. Table S6. SNPs by common gene. Table S7. replicated SNPs. Table S8. GWAS-SNP by ancestry. Table S9. SNP clusters. Table S10. unique LD pairs. Table S11. SNP LD groups. Table S12. high LD groups. Table S13. PRISMA Checklist.

## Abbreviations

25OHD, 25-hydroxyvitamin D; DBP, vitamin D-binding protein; GWAS, genome-wide association study; LD, linkage disequilibrium; MAF, minor allele frequency; PRISMA, Preferred Reporting Items for Systematic Reviews and Meta-Analyses; Q-Genie, Quality of Genetic Association Studies; RINT, rank-based inverse normal transformation; SNP, single nucleotide polymorphism; VDD, vitamin D deficiency.
